# Comparison of Diazotrophic Composition and Distribution in the South China Sea and the Western Pacific Ocean

**DOI:** 10.3390/biology10060555

**Published:** 2021-06-20

**Authors:** Changling Ding, Chao Wu, Liuyang Li, Laxman Pujari, Guicheng Zhang, Jun Sun

**Affiliations:** 1 Research Centre for Indian Ocean Ecosystem, Tianjin University of Science and Technology, Tianjin 300457, China; dingcl0405@163.com (C.D.); wuchao@tust.edu.cn (C.W.); liuyangli@sjtu.edu.cn (L.L.); marine.laxman1@gmail.com (L.P.); pigment@tust.edu.cn (G.Z.); 2 College of Marine Science and Technology, China University of Geosciences (Wuhan), Wuhan 430074, China

**Keywords:** diazotroph, Proteobacteria, *Trichodesmium*, unicellular cyanobacteria, South China Sea, Western Pacific Ocean

## Abstract

**Simple Summary:**

Diazotrophs are important contributors of bioavailable nitrogen that is essential to maintaining biological productivity in marine ecosystems. In this study, surface water samples were analyzed to explore the spatial variation of the diazotrophic community in the upper seawater of the South China Sea (SCS) and the Western Pacific Ocean (WPO). The well-conserved gene *nifH*, which is considered an important marker gene, was used for analyzing the phylogeny, diversity, and abundance of diazotrophs in this study. Our results showed that Proteobacteria was the main diazotroph in the SCS, while Cyanobacteria accounted for the largest proportion in the diazotroph community in the WPO. In addition, high abundances of diazotrophs in the subequatorial WPO indicated the occurrence of diazotrophs blooming. Variation in the composition of diazotrophs was significantly correlated with temperature, dissolved inorganic nitrogen, dissolved inorganic phosphate, and spatial variables between these regions. Our results provide insights into the ecological success and biogeochemical importance of diazotrophic communities in changing environments.

**Abstract:**

The variation of diazotrophs has been elusive in multiple SCS and WPO regions due to insufficient data. Therefore, the dynamics of diazotrophic composition and distribution were investigated in this study, based on high-throughput sequencing and quantitative PCR of the *nifH* gene. We found that Proteobacteria dominated the diazotrophic community in the river-impacted SCS and cyanobacteria and Proteobacteria were more abundant in the ocean-dominated SCS and WPO. The qPCR analysis showed that cyanobacterial *Trichodesmium* was abundant in the Pearl River plume and in the SCS basin influenced by the Kuroshio intrusion, and it also thrived in the subequatorial region of the WPO. Unicellular cyanobacteria UCYN-A were mainly detected in the river-impacted area, UCYN-B was abundant in the WPO, UCYN-C had a relatively high abundance in the ocean-dominated area, and a preponderance of γ-Proteobacteria γ-24774A11 was observed in the ocean-dominated SCS and pelagic WPO. Diazotrophic communities had significant distance–decay relationships, reflecting clear biogeographic patterns in the study area. The variations of diazotrophic community structure were well explained by dissolved inorganic nitrogen, dissolved inorganic phosphate by an eigenvector spatial variable PCNM1. These results provide further information to help determine the ecological mechanism of elusive diazotrophic communities in different ocean ecosystems.

## 1. Introduction

The South China Sea (SCS) has an extensive basin and broad continental shelves. With nutrients sourced from land and open ocean, the entire SCS can be divided into two distinct areas, river-dominated ocean margin and ocean-dominated margin, respectively [1]. The wind-induced circulation and mesoscale eddies predominantly impact biogeochemical progress in the upper ocean SCS [2,3], and the riverine input from the Pearl and Mekong Rivers dramatically affects nutrient availability in the SCS [4,5]. The SCS is connected to the open ocean through a major channel, the Luzon Strait, which allows effective water exchange with the Western Pacific Ocean (WPO). The upper mixing in the SCS is a significant process that drives the water exchange between the SCS and WPO. The intrusion of the Kuroshio is a key factor that contributes to the enhanced upper mixing in the northern SCS, when the Kuroshio current from the WPO passes the Luzon Strait [6]. The WPO is subject to western boundary currents (WBC) containing multiple currents and eddies [7]. The complex environment with a permanently stratified water column, nutrient-laden river transport, and oligotrophic and pelagic basin provide distinctive habitats for the development of diverse microbial communities [8]. In the surface water of the pelagic SCS, the nitrogen limitation caused by stratified and oligotrophic environments favors the growth of N_2_-fixer diazotrophs [9], which in turn provides bioavailable nitrogen to N-starving ecosystems [10,11]. The Pacific contributes to the most favorable habitat for diazotrophs due to the oligotrophic environment [12]. Field studies have highlighted that N_2_ fixation by the diazotrophs can support 50% or more “new production” in the oceanic waters [13].

Previous studies in the SCS have shown that the filamentous cyanobacteria *Trichodesmium* are the major group of diazotrophs [14,15], while *Calothrix rhizosoleniae* and *Richelia intracellularis* were sparsely distributed [16]. With the development of molecular techniques in marine biology [17,18,19], the well-conserved *nifH* gene, which encodes the iron protein component of the nitrogenase complex in diazotrophs, has been the focus of phylogenetic and ecological studies in oceanic habitats [20,21,22,23,24]. Five major phylogenetic *nifH* clusters have been described, among which Cyanobacteria as well as α-, β-, and γ-Proteobacteria categorized into cluster I are the most popular putative diazotrophs in marine environments [25,26]. Many observations based on frequent retrievals of *nifH* gene sequences, suggest that unicellular cyanobacteria and heterotrophic diazotrophs are in equal or greater abundances than filamentous cyanobacteria, both in the SCS and the Pacific Ocean [13,27,28,29,30,31,32,33,34,35,36]. Diazotrophs have a variable geographic distribution due to individual physiological and ecological constraints [37,38]. Published studies showed that α-Proteobacteria and *Trichodesmium* were dominant in the southern SCS [33], while non-cyanobacteria, γ-Proteobacteria, and α-Proteobacteria formed the dominant diazotrophic group in the northern SCS [30,34]. Recently, *Trichodesmium* was observed to be the dominant genus in the SCS and western equatorial Pacific, whereas the unicellular cyanobacterium UCYN-B dominated in the Philippine Sea [36]. In contrast, unicellular cyanobacteria together with γ-Proteobacteria γ-24774A11 made up the majority of the diazotrophic community in the North Pacific [13].

Many studies have been hampered by the need to create large clone libraries to characterize true sequence diversities and were also limited at several sampling sites or sections. Furthermore, the spatial relationships of the diazotrophic communities between the shelf SCS, central SCS, and pelagic western boundary of the Pacific Ocean (WPO) were explained insufficiently. Moreover, the variation of the diazotrophic communities is unknown between the river-dominated ocean margin and the ocean-dominated margin, and the connectivity relationship of the diazotrophic communities between the SCS and western Pacific is elusive. In this study, large-scale surveys were carried out across the continental region and the vast basin of the SCS, extending to the WPO where it is influenced by WBC. The first detailed investigation of the diazotrophic communities was conducted in the central SCS and the WPO. Additionally, high-throughput sequencing technology and quantitative PCR (qPCR) were adopted to detect the phylogenetic diversity and structure of the diazotrophic communities. This study aimed to (1) characterize the diversities and heterogeneities of the diazotrophic communities in the SCS and WPO, (2) elucidate the environmental factors that influence the structure and spatial distribution of diazotrophic communities in the marginal sea and oceanic ecosystems, and (3) provide insights into the ecological success and biogeochemical importance of the diazotrophic communities in changing environments.

## 2. Materials and Methods

### 2.1. Study Area

**Figure 1:**
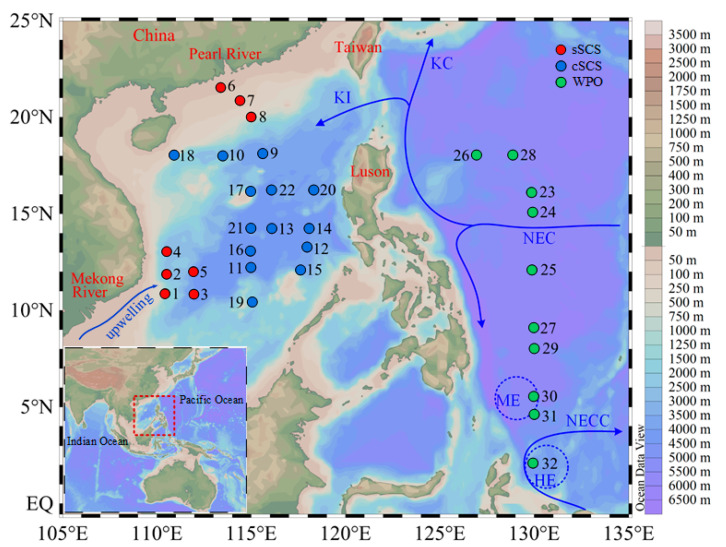
Areas and sampling areas investigated in this study. The schematic of the major surface currents shown refers to Hu et al. (2015). KI, Kuroshio Intrusion; KC, Kuroshio Current; NEC, North Equatorial Current; NECC, North Equatorial Counter Current; HE, Halmahera Eddy; ME, Mindanao Eddy.

A total of three cruises were carried out, one to the marginal SCS onboard R/V “Shiyan 3” from September to October 2016, one to the central SCS onboard R/V “Shiyan 1” from March to May 2017, and one to the WPO onboard R/V “Kexue” from October to November 2017 (Figure 1). The study area extended from the northern continental shelf SCS (sSCS) to the central SCS (cSCS), and to the WPO. The marginal SCS was bordered by China on the north and Vietnam on the west. The northern region referred to one transect from the Pearl River plume to oceanic waters in the northern SCS, where the runoff from the Pearl River carries a large quantity of fresh water and dissolved nutrients. The western region was bordered by the steep slopes of the Vietnamese coast and beyond to stations extending to a depth of 4000 m. The northward flow of Vietnam’s offshore waters in summer causes a local increase of upwelling in Vietnam with an intensity between 12° N and 13° N [39]. The central SCS covered the vast basin with a maximum depth that reaches 4700 m, where the central gyre is permanently stratified and oligotrophic [9]. The Kuroshio Intrusion (KI) with warm, saline, and oligotrophic properties through the Luzon Strait affected the circulation and water chemistry of the northern boundary of the basin [40]. The WPO region was strongly controlled by the western boundary current systems (WBCs), which form the Kuroshio Current (KC) northwards and the Mindanao Current (MC) southwards. Concurrently, the anticyclonic Halmahera Eddy (HE) and the cyclonic Mindanao Eddy (ME) exist, respectively, in the north and south of the North Equatorial Counter Current (NECC) derived from the southward MC. The water mass of HE and ME originate from the Northern Pacific tropical water and Southern Pacific tropical water, respectively [7].

### 2.2. Sampling and Physicochemical Analysis

Surface water samples for DNA analysis and the determination of related biogeochemical parameters were collected with a Niskin bottle rosette (12-L) equipped with a Conductivity-Temperature-Depth sensor (CTD, SBE 9/11 plus, SeaBird Inc., Bellevue, WA, USA) from 32 sampling sites at a 5 m depth among the three defined regions, namely sSCS, cSCS, and WPO. Collected seawater samples were transferred into a 15 L HCl-rinsed bucket for further subsampling. Subsamples for nutrient analysis were transferred into 100 mL HCl-rinsed bottles and immediately stored at −20 °C until analyzed in the laboratory. For chlorophyll *a* (Chl *a*) analysis, 500 mL or 1 L samples from each station were vacuum filtered (<10 mm Hg) through 25 mm Whatman GF/F filters and stored in the dark at −20 °C until analyzed in the laboratory. For DNA samples, 1–4 L seawater from each station was filtered (<10 mm Hg) through 0.22 μm GTTP filters (Millipore, Billerica, MA, USA) using gentle peristaltic pumping. All filters were immediately frozen and stored in liquid nitrogen until ready for DNA extraction. Additionally, temperature, salinity, and depth data were obtained from the CTD profiler.

After each cruise, the Chl *a* samples were analyzed immediately in the laboratory. The Chl *a* filters were extracted with 90% acetone. Extracts were then refrigerated at 4 °C for 24 h, after which Chl *a* concentrations were determined using a Trilogy (CHL NA, Model # 046) fluorometer (Turner Designs, San Jose, CA, USA). Nutrient concentrations including nitrate ( ${\mathrm{NO}}_{3}^{-}$), nitrite (${\mathrm{NO}}_{2}^{-}$), phosphate (${\mathrm{PO}}_{4}^{3-}$), silicate (${\mathrm{SiO}}_{3}^{2-}$), and ammonium (${\mathrm{NH}}_{4}^{+}$) were measured using a Technicon AA3 Auto-Analyzer (Bran + Luebbe, Norderstedt, Germany) based on classical colorimetric methods. Concentrations of ${\mathrm{NO}}_{3}^{-}$ and ${\mathrm{NO}}_{2}^{-}$ were measured using copper-cadmium column reduction methods, whereas concentrations of ${\mathrm{PO}}_{4}^{3-}$, ${\mathrm{SiO}}_{3}^{2-}$, and ${\mathrm{NH}}_{4}^{+}$) were measured by the phosphor-molybdate complex method, the silico-molybdate complex method, and the indophenol blue method, respectively [41].

### 2.3. DNA Extraction, PCR Amplification, and Sequencing

Genomic DNA was extracted using DNeasy PowerWater^®^ Kit (Qiagen, Hilden, Germany) following the manufacturer’s instructions. Note that the solutions used in our experiment were all molecular grade. The quality and quantity of extracted DNA were assessed using a Nanodrop spectrometer (Thermal Scientific, Wilmington, DE, USA), and extracts were stored at −20 °C until further processing. Nested polymerase chain reaction (nested PCR) was performed to amplify the fragments of *nifH* genes from the genomic DNA following the protocol outlined in Zehr et al. (1998) [18]. PCRs were conducted in quintuplicate using a Veriti 9902 thermocycler (Applied Biosystems, Foster City, CA, USA). Each reaction volume of 10 μL contained 1 × PCR buffer, 4 mM of MgCl_2_, 400 mM of dNTPs, 1 μM each of forward and reverse primers (*nifH*3 and *nifH*4 for primary, *nifH*1 and *nifH*2 for secondary PCR), 0.2-unit KOD FX Neo polymerase (Toyobo, Osaka, Japan), and 1 μL of template DNA (genomic DNA for the first round, and PCR products from the primary PCR for the second round). Negative controls were also prepared in our study by replacing template DNA with nuclease-free water. To distinguish samples after sequencing, 7-base barcode sequences were attached to the 5′ of the *nifH*3 or *nifH*4 primers. The thermal profile used for the *nifH* gene amplification in the present study was consistent with that in a previous report by Wu et al. (2019) [24]. PCR products were checked using 1.8% agarose gel electrophoresis (BioWest, Castropol, Spain) after amplification, and products with clear bands (approximately 360 bp bands) and simultaneously negative controls with no visible bands were considered suitable for further sequencing. Quintuplicate PCR products were pooled in equal amounts and purified via an Invitrogen PureLink^®^ Quick Gel Extraction Kit (Invitrogen, CA, USA) following the manufacturer’s instructions. The libraries were constructed and sequenced via paired-end chemistry (PE300) on an Illumina Miseq platform (Illumina, San Diego, CA, USA) at Allwegene Technologies, Beijing, China.

### 2.4. Quality Control and Analysis of Sequencing Data

After sequencing, the raw sequencing data and their corresponding sequencing quality were obtained from an Illumina Miseq platform via base calling. The raw sequencing reads were first demultiplexed and quality filtered by their barcode sequences, permitting up to one mismatch. Quality control and sequencing data analyses were performed using the open-source software pipeline QIIME [42]. Paired-end reads were merged into full-length sequences by FLASH v1.2.7 software [43]. The minimal overlapping length was 10 bp, and the maximum mismatch ratio was 0.2. Paired-end reads without overlaps were removed from the pool. The merged full-length sequences were quality filtered by removing sequences less than 300 bases, sequences containing homopolymers (homopolymers ≥ 8 bases), and sequences containing ambiguous bases [44]. The chimera sequences were also removed from the raw tags by comparing tags with the reference database in the UCHIME v4.2 software, and the remaining effective tags were grouped into operational taxonomic units (OTUs) with 97% similarity using USEARCH v10.0 [45]. In the present study, the most common sequences in each OTU were selected as representative sequences. Rarefaction curves were calculated using the Past v3.0 software and plotted using Origin 2020 software based on the OTU table [24].

To taxonomically classify the OTUs, representative sequences were first translated into amino acid sequences and the protein sequences database in the National Center for Biotechnology Information (NCBI) databases was searched using BLASTX [46]. The most closely related sequences (>96% similarly) were chosen as alignment sequences. Finally, the representative sequences and alignment sequences were aligned with Clustal W in MEGA X [47], and a phylogenetic neighbor-joining tree was subsequently constructed using the maximum likelihood method. The LG+G model was selected after model evaluation, bootstrap values were determined by resampling 1000 times, and bootstrap values greater than 50% were shown near the nodes. The constructed tree was further edited by Interactive Tree of Life (iTOL), an online tool for managing phylogenetic trees [48]. The raw sequencing data for the *nifH* gene has been submitted to the NCBI Sequence Read Archive (SRA) with accession number SUB7406573 (https://submit.ncbi.nlm.nih.gov/subs/sra/SUB7406573/overview, accessed on 13 May 2020).

### 2.5. Quantification of Main Cyanobacterial nifH Phylotypes

To provide insights into the potential roles of diazotroph groups in local environments, the abundances of representative diazotrophic phylotypes referring to *Trichodesmium*, UCYN-A, UCYN-B, UCYN-C, and γ-24774A11 were determined using quantitative polymerase chain reaction (qPCR) targeting the *nifH* gene. Besides, a genus that is identical to *Sagittula castanea* was also selected for qPCR analysis. *Sagittula castanea* belonged to α-Proteobacterial diazotroph, it is an important member of the *Roseobacter* group. Quantifications of targeted diazotrophs were conducted using an ABI Step One Plus Real-Time PCR System (Applied Biosystems, Foster City, CA, USA). The corresponding *nifH* standards were obtained from the clone library of environmental samples, except for UCYN-C and *Sagittula castanea*, for which the specific primers were used to directly amplify the environmental samples. The specific primers, probes, and standard clones used in the present study are described in Table 1. The qPCR reactions were performed in duplicate in a final volume of 10 μL, containing 5 μL of 2 × Premix Ex Taq ^TM^ (Takara Bio, Tokyo, Japan), 0.2 μL of 50 × ROX Reference Dye, 0.4 μL of 10 μM forward and reverse primers, 0.4 μL of TaqMan probe, 1 μL of template DNA, and the remaining volume made up of nuclease-free water. The qPCR reaction mixture was denatured at 95 °C for 30 s, followed by 45 cycles of denaturation at 95 °C for 5 s, and annealing at 60 °C for 30 s. Standard curves were constructed from 10-fold dilution series based on 10 to 107 gene copies per reaction. Linear regression (R^2^) values and amplification efficiencies (E) of each standard curve greater than 0.99 and 90%, respectively, were considered effective. The amplification efficiency was calculated by the equation E = 10^−1/m^ − 1, where m is the slope of the standard curve. Non-target templates were also tested under the same conditions as were used for the standards and samples. Where amplification of non-target templates occurred (Ct values ranged from 35 to 38), the non-target template gene copies were subtracted from the sample values to adjust for the slight contamination.

### 2.6. Analysis of Community Composition and Diversity

Diazotrophic community structures and boxplots of representative diazotroph abundances were plotted using R.3.6.1 software [49]. To compare the relative composition of diazotrophic communities, alpha diversity and beta diversity were calculated based on the OTU tables. Alpha diversity indices, including richness, Shannon index, Pielou index, and Chao1 richness estimator were calculated using the R v3.6.1 software with reference to R Development Core Team (2013) [50]. The relative contribution of the dominant taxa of diazotrophs was determined using the R v3.6.1 software and was applied to calculate the intersections of OTUs [49]. Non-metric multidimensional scaling (NMDS) analysis of diazotrophic communities was performed using Past3 software to characterize the horizontal distribution patterns of diazotrophic communities (http://www.canadiancontent.net/tech/download/PAST.html, accessed on 13 May 2020). In addition, station maps and distribution patterns of representative diazotrophs were plotted using the Ocean Data View (ODV) software [51].

### 2.7. Statistical Analysis of Community Composition, Environmental Variables, and Geographical Distance

The Kruskal–Wallis test was performed for the intergroup statistical analysis of environmental factors, alpha diversity indices, and diazotroph gene copies using the vegan package [52]. The distance–decay relationship was analyzed to explore the biogeography patterns of diazotrophic communities, explained by the relationship between community similarity and the longitude and latitude coordinates of each sampling site using the R v3.6.1 software [53]. The principal coordinates of neighbor matrices (PCNM) analysis, based on geographic coordinates, was used to examine spatial structures of diazotrophic communities [54]. Using classical forward selection, the eigenvectors PCNM1, PCNM3, and PCNM5 (R^2^ = 0.165) were chosen for further analysis (Table S1). To explore the potential controlling factors of the composition of the diazotrophic communities, the Mantel test and Spearman’s correlation analysis were computed among the abundance of diazotrophic group OTUs, qPCR quantifies, and environmental factors using the R v3.6.1 software [50]. Abundances of diazotroph OTUs and environmental parameters were used for Transformation-based redundancy analysis (tb-RDA) using the phyloseq package [55]. In detail, to test the collinearity between environmental factors, the variance inflation factor (VIF) value was calculated using the “vegan” package. The environmental factors with a VIF value of greater than 10 were rejected. Then effective data were selected for further analysis. The significance of each response variable was confirmed with an analysis of variance (ANOVA) for tb-RDA, only significant (*p* value < 0.05) response variables were kept in the model [52].

## 3. Results

### 3.1. Environmental Conditions

**Figure 2:**
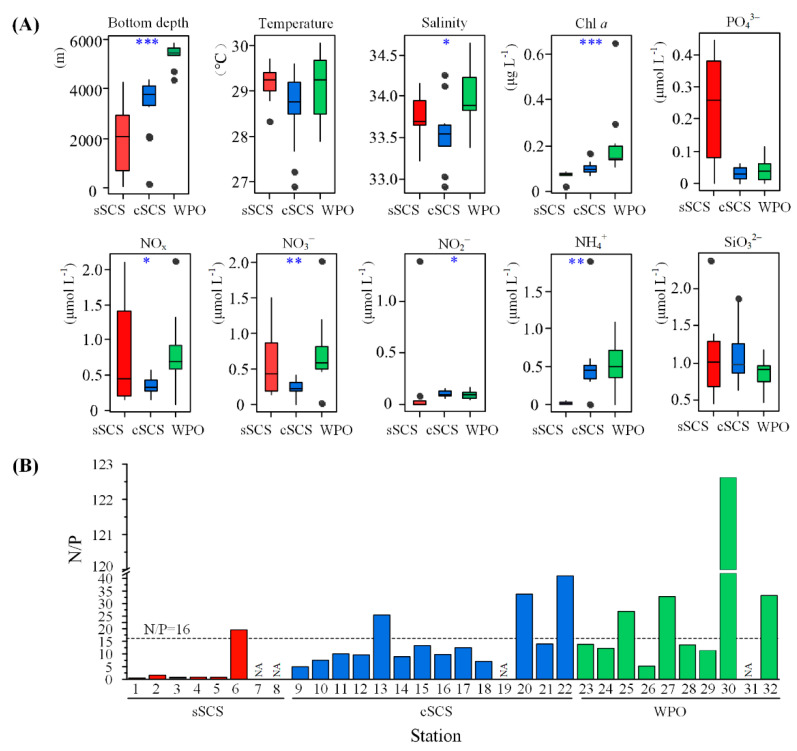
Environmental parameters values and differences in three regions (**A**) (* *p* < 0.05; ** *p* < 0.01; *** *p* < 0.001, Kruskal–Wallis test). The ratio of NO_x_ to ${\mathrm{PO}}_{4}^{3-}$ (N/P) concentration was shown in the bar diagram (**B**) (dashed line means N/P = 16). NA means no data result from the ${\mathrm{PO}}_{4}^{3-}$ concentration below the limit of detection (<0.024 uM).

The relationship between the surface water environmental parameters of different regions is shown in Figure 2. The sea surface temperature (SST) varied from 28.34 to 29.72 °C, 26.89 to 29.59 °C, and 27.91 to 30.05 °C in the sSCS, cSCS, and WPO, respectively. The SST of the cSCS was lower than that in other regions. However, there were no significant differences between the SSTs for these regions. The sea surface salinity (SSS) ranged from 26.40 to 34.16, 32.91 to 34.35, and 33.39 to 34.64 in the sSCS, cSCS, and WPO, respectively. As shown in Figure 2A, there were significant differences between SSSs for these regions (*p* < 0.05). The surface Chl a concentration ranged from 0.02 to 1.68 μg L^−1^, 0.07 to 0.17 μg L^−1^, and 0.11 to 0.65 μg L^−1^ in the sSCS, cSCS, and WPO, respectively. The maximum concentration of Chl *a* was 1.68 μg L^−1^ at Stn.6 near the Pearl River plume in the SCS, followed by 0.65 μg L^−1^ at Stn.30 in the WPO. Notably, the surface Chl *a* concentrations were significantly different between the SCS and WPO (*p* < 0.001). For surface nutrition, the concentration of SiO2-3 ranged from 0.44 to 1.88 μmol L^−1^, 1.25 to 2.38 μmol L^−1^, and 0.46 to 1.17 μmol L^−1^ in the sSCS, cSCS, and WPO, respectively; there were no significant differences between SCS and WPO. There were significant differences in nutrients concentrations, except for ${\mathrm{SiO}}_{3}^{2-}$ and ${\mathrm{PO}}_{4}^{3-}$, between these regions. Concentrations of NO_x_, representing the sum of ${\mathrm{NO}}_{3}^{-}$ and ${\mathrm{NO}}_{2}^{-}$, were lower in the cSCS (ranging from 1.35 to 2.10 μmol L^−1^) than that in the sSCS and WPO regions (ranging from 0.14 to 0.60 μmol L^−1^ and 0.08 to 2.11 μmol L^−1^, respectively). The concentration of ${\mathrm{PO}}_{4}^{3-}$ was higher in the sSCS (ranging from 0.01 to 0.44 μmol L^−1^), compared with that in the cSCS (ranging from 0.01 to 0.06 μmol L^−1^) and WPO (ranging from 0.01 to 0.12 μmol L^−1^). For N/P ratio, the bar plot clearly showed that almost all data points occurred below N/P Redfield ratio line in the sSCS and cSCS, especially at lower levels in the sSCS (dashed line, Figure 2B). While data points with a relatively higher ratio in the WPO evenly distributed on both sides of the reference line, in particular, the N/P ratio was detected up to 122.62 in Stn.30. In addition, bottom depth varied significantly between study areas (*p* < 0.001), revealing geographical differences.

### 3.2. Sequencing Statistics and Diversity Estimates

**Figure A1:**
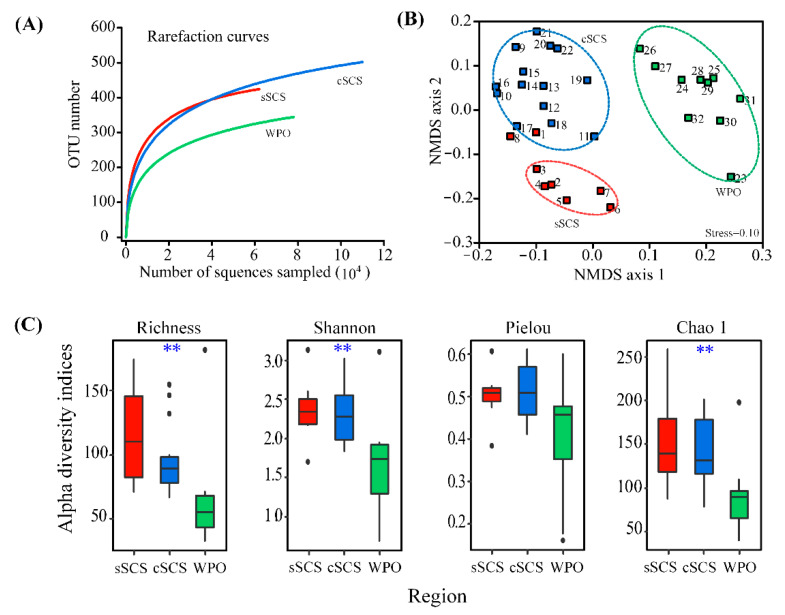
The rarefaction curves plateau (**A**) was calculated in the study. Non-metric multidimensional scaling (NMDS) analysis of diazotrophic communities in different stations (**B**), different groups were circled with dotted lines (sSCS, cSCS, WPO). Diversity and predicted richness of genetic community, and the Kruskal–Wallis tests of alpha diversity indices among the study areas (**C**). (** *p* < 0.01).

The results from the high-throughput sequencing showed that the sequence numbers ranged from 7959 to 80,504; a total of 81,7548 effective tags were obtained after performing quality control on all samples. After resampling, 7959 sequences were obtained in each sample. Based on a similarity of 97%, 927 OTUs were recruited in all samples. The rarefaction curve plateaus are shown in Figure A1. The sequencing coverage (C) was above 98% in the rarefaction analysis, suggesting the sequencing effort was sufficient to represent *nifH* gene diversity. The OTU counts ranged from 71 to 174, 67 to 154, and 33 to 181 in the sSCS, cSCS, and WPO, respectively. Our data indicated that the OTU counts in the SCS were higher than those in the WPO. The NMDS analysis presented a regional separation between samples collected from the WPO, sSCS, and cSCS (Figure A1). Alpha diversity was estimated by the Richness, Shannon, Pielou, and Chao1 indexes. The Richness index was used to determine the number of species observed (OTUs). The Shannon index was used to describe the biodiversity of an ecological region. The Pielou index was chosen to estimate the evenness of different species (OTUs) in the community. The Chao1 index was used to estimate the total number of species (OTUs). In this study, there were significant differences in the indices of Richness, Shannon, and Chao1 between the three regions (*p* < 0.01) (Figure A1). High diversity indices were observed in the sSCS samples, but, in contrast, relatively low diversity indices were observed in the WPO samples.

### 3.3. Phylogeny and Composition of Diazotrophic Communities

**Figure A2:**
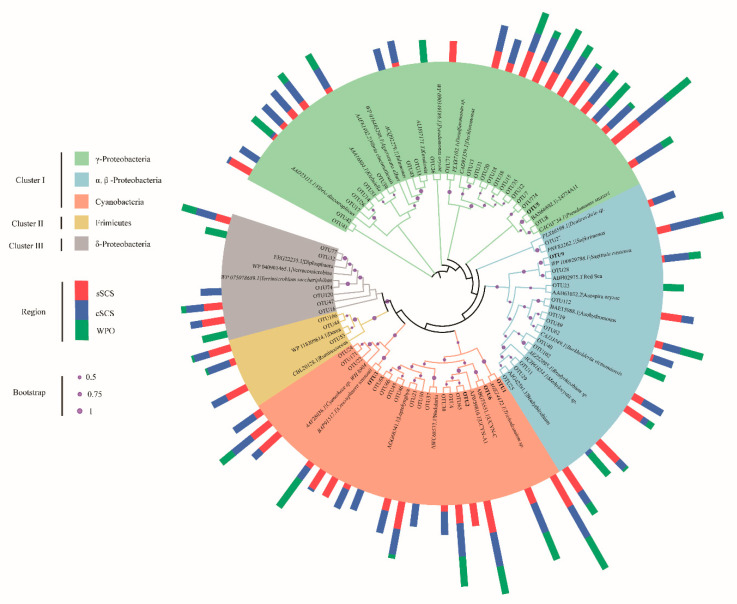
Maximum-likelihood phylogenetic tree is structured with *nifH* amino acid sequences in this study. Representative OTU sequences were indicated in boldface. The multi-bar shows the relative abundances of OTU sequences (log10) from different regions. Bootstrap resampling was performed 1000 times and its values higher than 50% are shown.

This study only focused on the top OTUs because most of the OTUs were unidentified. The OTUs containing more than 0.1% individual sequences were defined as top OTUs in this study. A total of 62 OTUs were selected for subsequent phylogenetic analysis. The top 62 OTUs corresponded to lineages within three clusters of *nifH* genes. By aligning with the reference sequence from NCBI, 54 OTUs belonged to the largest group cluster I including Cyanobacteria and α-, β-, and γ-Proteobacteria. A total of 3 OTUs belonged to cluster II (*Clostridia* spp.), and 5 OTUs belonged to cluster III (δ-Proteobacteria) (Figure A2). In total, OTUs affiliated with Proteobacteria accounted for more than half counts, including γ-Proteobacteria (23/62 OTUs) and α-, β-Proteobacteria (13/62 OTUs), followed by Cyanobacteria (17/62 OTUs) and other groups (9/62 OTUs).

The abundances of OTUs clustered with Cyanobacteria were considerably higher than other phyla, followed by γ-Proteobacteria and α-, β-Proteobacteria. The average relative proportion of Cyanobacteria in the community contributed to 53.27%, 55.27%, and 72.12% in the sSCS, cSCS, and WPO samples, respectively. Among which, abundant genus OTU 1 shared 100% similarity with *Trichodesmium* (Cyanobacteria) were recovered from all surface waters (Figure A2), accounting for 34.26% (sSCS), 21.14% (cSCS), and 37.97% (WPO) in total top OTUs. Particularly at Stn 27, Stn 31, and Stn 32, relative abundances of OTU 1 were high up to 88.60%, 70.13%, and 86.48%, respectively. Furthermore, OTU 2, OTU 3, and OTU 6 shared 100% similarity, respectively, with UCYN-A (Cyanobacteria), *Crocosphaera watsonii* (UCYN-B, Cyanobacteria), and UCYN-C (Cyanobacteria), which were also typically abundant genera in the survey area. OTU 2 (UCYN-A) were mainly observed in the sSCS (average 11.69%) and cSCS (average 12.53%), the relative abundance of which was low in the WPO (4.08%). Furthermore, OTU 4, OTU30, and OTU65 clustered with UCYN-A were recovered sporadically in the survey (Figure A2). The relative abundance of OTU 3 (UCYN-B) was high in the WPO (average 11.69%) and was recovered sporadically in other regions. The relative abundances of OTU 6 (UCYN-C) in the cSCS (average 5.30%) and the WPO (average 4.39%) were both an order of magnitude higher than that in the sSCS (average 0.13%). In addition, symbiont *Richelia intracellularis* (OTU 100) was annotated in this study, the proportion of which was only 0.06% in the survey.

**Figure 3:**
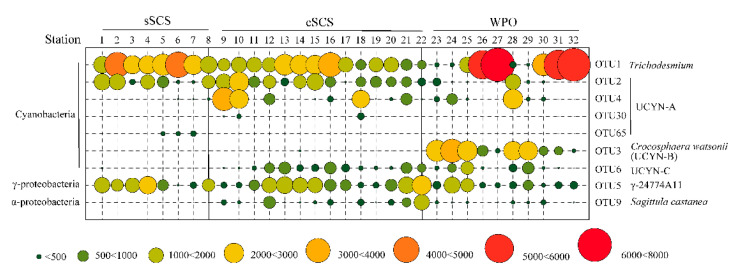
The relative contribution of dominant taxa of diazotrophs in study stations (OTU numbers).

**Figure A3:**
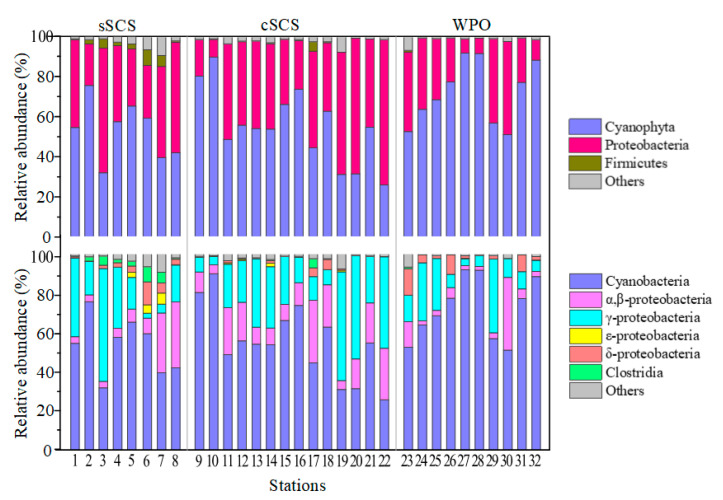
The relative abundances (%) of diazotroph groups at sampling stations.

For OTUs clustered with Proteobacteria, abundances of γ-Proteobacteria OTUs were comparable between the three regions, which contributed 23.59%, 25.62%, and 14.78% on average in the sSCS, cSCS, and WPO samples, respectively. Abundances of α-, β-Proteobacteria OTUs (average 7.5%) in the WPO samples were relatively low compared with that in the sSCS (average 11.70%) and the cSCS (average 15.41%) (Figure A3). Abundances of the rest phyla OTUs accounted for a small proportion of the total, which was 11.44%, 3.69%, and 7.50%, respectively. OTU 5 shared 100% similarity with γ-24774A11 (γ-Proteobacteria), which were the predominant groups within the Proteobacteria cluster and were more abundant in the cSCS (average 13.25%) and sSCS (average 13.76%) compared with that in the WPO (average 7.53%). OTU 9 had 100% similarity with the recently isolated species, *Sagittula castanea* (Rhodobacteraceae, α-Proteobacteria), and its abundance decreased orderly in the cSCS (average 4.13%), the WPO (average 0.87%), and the sSCS (average 0.07%). In brief, sequences associated with *Trichodesmium*, UCYN-A, *Crocosphaera watsonii* (UCYN-B), UCYN-C, and γ-24774A11 were relatively popular groups in the three regions (Figure 3).

### 3.4. Quantification of Representative Diazotrophs

**Figure 4:**
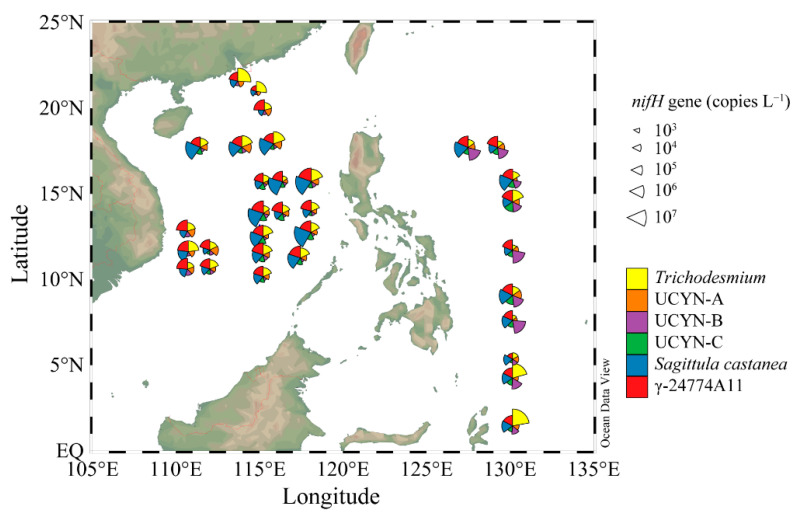
The *nifH* gene copies and distributions of representative genera phylotypes in different regions.

The *nifH* sequences of six representative diazotrophic phylotypes, *Trichodesmium,* UCYN-A, *Crocosphaera watsonii*, UCYN-C, *Sagittula castanea*, and γ-24774A11, were selected for quantitative analysis by qPCR. *Trichodesmium* was detected in the highest abundance at Stn.32 (2.64 × 10^7^ copies L^−1^) in the WPO, and was also present in high abundance (3.71 × 10^6^ copies L^−1^) at Stn.6 of the sSCS. The distribution patterns for these phylotypes showed that *Trichodesmium* mainly thrived in the subequatorial area of the WPO, followed by the Pearl River plume, and the northern edge of the cSCS (along 18° N) (Figure 4). In contrast, the abundance of *Trichodesmium* was much lower in the northern part of the WPO, and their abundance was relatively higher in the basin edge than in the central part in the cSCS.

**Figure A4:**
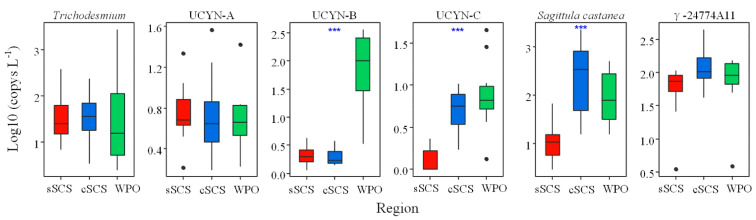
The *nifH* gene copies of representative genera phylotypes among different regions were analyzed by the Kruskal–Wallis tests (B) (*** *p* < 0.001).

The abundance of UCYN-A ranged from 0.65 × 10^4^ to 2.09 × 10^5^ copies L^−1^, 0.57 × 10^4^ to 3.59 × 10^5^ copies L^−1^ and 0.69 × 10^4^ to 2.53 × 10^5^ copies L^−1^ in the sSCS, cSCS, and WPO, respectively (Figure 4). The abundance of *Crocosphaera watsonii* (UCYN-B) ranged from 2.45 × 10^4^ to 3.52 × 10^6^ copies L^−1^ in the WPO, and was significantly higher than that in the two SCS regions (*p* < 0.001, Kruskal–Wallis tests) (Figure A4), with abundances ranging from 0.16 × 10^4^ to 3.13 × 10^4^ copies L^−1^ and 0.40 × 10^4^ to 2.17 × 10^4^ copies L^−1^ in the sSCS and cSCS, respectively. For UCYN-C, the abundance of this group ranged from 0.63 × 10^4^ copies L^−1^ to 1.73 × 10^4^ copies L^−1^ in the sSCS, 0.71 × 10^4^ copies L^−1^ to 9.19 × 10^4^ copies L^−1^ in the cSCS, and 0.34 × 10^4^ copies L^−1^ to 4.43 × 10^5^ copies L^−1^ in the WPO. Similarly, the abundances of UCYN-C showed significant regional variations (*p* < 0.001, Kruskal–Wallis tests) (Figure A4). The distribution patterns of these three unicellular cyanobacteria groups also showed regional differences. UCYN-A was present in high abundance in the western part, near the coast of Vietnam, and in the northern boundary of the cSCS. *Crocosphaera watsonii* was abundant in the WPO but sparse in the SCS, whereas UCYN-C was mainly distributed in the cSCS basin and in the open ocean of the WPO (Figure 4).

The Proteobacterial taxa *Sagittula castanea* and γ-24774A11 were prevalent in the present study. *Sagittula castanea* was abundant in the cSCS region, with abundances ranging from 1.95 × 10^4^ to 2.25 × 10^7^ copies L^−1^ (*p* < 0.001, Kruskal–Wallis tests) (Figure A4). In contrast, the abundances of *Sagittula castanea* were relatively low in the sSCS (ranged from 1.95 × 10^4^ to 6.57 × 10^5^ copies L^−1^) and in the WPO (ranged from 1.47 × 10^4^ to 4.93 × 10^6^ copies L^−1^). A relatively low abundance of γ-24774A11 was detected in the sSCS region, with abundances ranging from 2.43 × 10^4^ to 1.05 × 10^6^ copies L^−1^. In addition, the abundance of γ-24774A11 in the cSCS was comparable to that in the WPO, with abundances ranging from 2.43 × 10^4^ to 4.50 × 10^6^ copies L^−1^ and 2.81 × 10^4^ to 1.53 × 10^6^ copies L^−1^ in the cSCS and WPO, respectively. The distribution patterns of *Sagittula castanea* were consistent with those of γ-24774A11, which were found to be mainly distributed in the basin with a bottom depth deeper than 2000 m rather than in continental areas (Figure 4).

### 3.5. Diazotrophic Communities in Relation to Spatial and Environmental Variables

**Figure 5:**
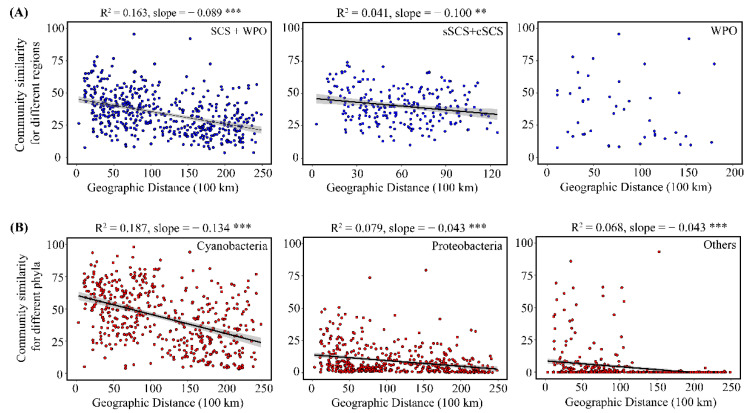
Community similarity for different regions (**A**) and Community similarity for different 810 phyla (**B**). (** *p* < 0.01; *** *p* < 0.001).

A plot of community similarity versus geographic distance for each sample revealed that the distance–decay curve was not significant at the WPO scale. In contrast, the diazotrophic community displayed a significant, negative distance–decay curve in the SCS (sSCS and cSCS, R^2^ = 0.041, slope = 0.100, *p* < 0.01), and the slope of this curve varied significantly between the three spatial scales (SCS and WPO, R^2^ = 0.163, slope = 0.089, *p* < 0.001) (Figure 5A). Furthermore, Cyanobacteria, Proteobacteria, and other phyla had significant distance–decay relationships in the overall sampling sites (*p* < 0.001), and the distance–decay slope within Cyanobacteria was steeper (R^2^ = 0.187, slope = 0.134) than other slopes within Proteobacteria (R^2^ = 0.079, slope = 0.043) and other phyla (R^2^ = 0.068, slope = 0.043) (Figure 5B). Overall, the similarity in diazotrophic community composition between the SCS and the WPO or the overall sampling sites decreased with increasing geographic distance.

**Figure 6:**
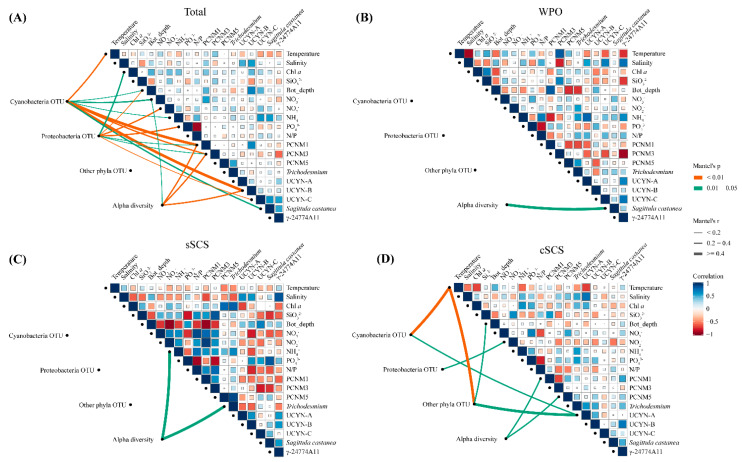
Mantel tests and Spearman’s correlation matrix among diazotrophic communities and environmental parameters in the total survey area (**A**), the WPO (**B**), the sSCS (**C**), the cSCS (**D**). Pairwise comparisons between environmental factors and gene copies of representative genera phylotypes (qPCR) are shown with a color gradient denoting Spearman’s correlation coefficient. The composition of diazotrophic communities (OTUs) was related to environmental parameters using Mantel tests to determine the Distribution of diazotrophs. Edge width corresponds to the Mantel’s r statistic for the corresponding correlations, and edge color denotes the statistical significance of Mantel’s p. The insignificant correlations were not be displayed.

**Figure A5:**
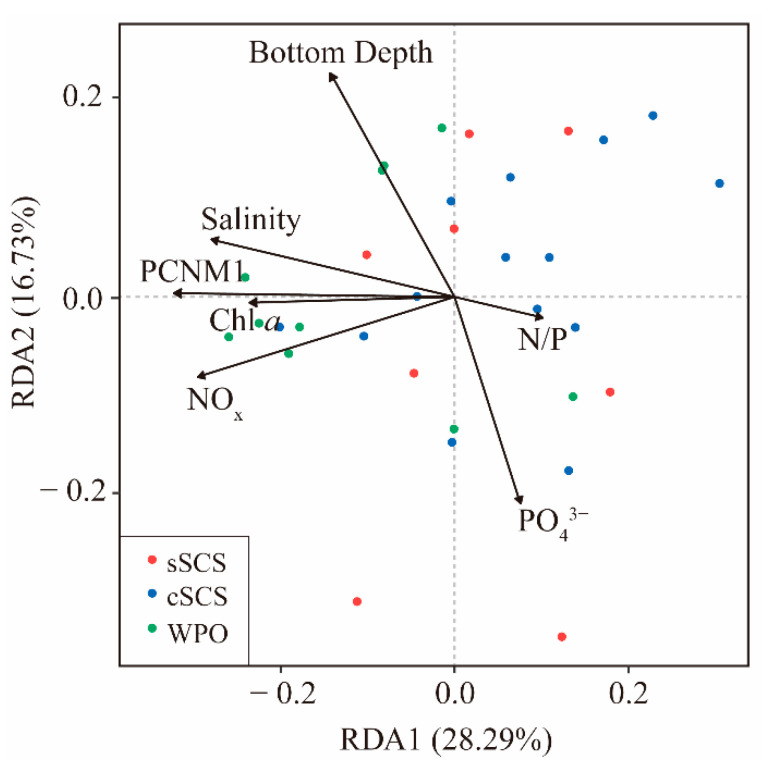
The transformation-based redundancy analysis (tb-RDA) is used to evaluate the effect of environmental fluctuations on the composition of diazotrophic communities in the SCS and the WPO.

To identify the relative importance of environmental factors contributing to the diazotrophic community composition, relationships between environmental factors, spatial variables, and relative abundances of diazotrophic groups and qPCR quantities of the representative genus were analyzed using Spearman’s correlation and Mantel’s tests (Figure 6). In the sSCS, qPCR quantities of *Trichodesmium* had a negative relationship with nitrogen nutrients, but a positive relationship with phosphorus nutrients. By contrast, the correlation between qPCR quantities of other diazotrophs and environmental factors had an inverse pattern. OTU abundances of diazotrophs were not significantly related to environmental factors and spatial variables, and ammonium salt had a statistically significant relationship with community diversity according to Mantel’s test (*p* < 0.05). In the cSCS, qPCR quantities of *Trichodesmium* and UCYN-A were negatively related to temperature and Proteobacterial *Sagittula castanea* and γ-24774A11 were negatively related to nitrogen nutrient. Moreover, temperature had a significant correlation with OTU abundances of Cyanobacteria and other phyla diazotrophs, inorganic nitrogen had a significant correlation with OTU abundances of Proteobacteria, and spatial variables were significantly correlated with community diversity. In contrast to the results obtained for the SCS, no significant correlation was observed between OTU abundances of diazotrophs and any environmental parameter in the WPO, whereas there was a negative relationship between temperature and qPCR quantities of diazotrophs except *Trichodesmium*, and a positive relationship between ammonium and qPCR quantities of all diazotrophs. In the entire study area, OTU abundances of Cyanobacteria had a significant correlation with temperature and spatial variables (PCNM1 and PCNM3) (*p* < 0.01), as well as ammonium and bottom depth (*p* < 0.05). OTU abundances of Proteobacteria was significantly related to bottom depth, nitrate, phosphate (*p* < 0.01), and Chl *a* concentration, spatial variables (PCNM1 and PCNM3) (*p* < 0.05). Furthermore, the diversity of the diazotrophic community had a significant relationship with nitrate (*p* < 0.01) and spatial variables (PCNM1 and PCNM3) (*p* < 0.05). The environmental sensitivity of the diazotrophic community was assessed by comparing how much of the compositional variation was explained by environmental parameters. Results from ANOVA for transformation-based redundancy analysis (tb-RDA) showed that the compositional variability of the whole diazotrophic community (OTUs) was well explained by NO_x_, ${\mathrm{PO}}_{4}^{3-}$, N/P ratio, salinity, Chl *a*, bottom depth, and PCNM1 (*p* < 0.05) (Figure A5).

## 4. Discussion

### 4.1. Role of Diazotrophs in the Nutrient-Replenished Marginal SCS

The upper ocean of the SCS has diverse ecosystems that are influenced by physical-biological oceanographic couplings. Nutrient concentrations combined with hydrological dynamics, including runoffs, cyclonic gyre, eddies, and upwellings shape the biotic community in the surface SCS. During this study, the highest nutrient concentration values but the lowest salinity were recorded for the northern sSCS (Stn.6), indicating that river input remarkably modulated the local ecological environment. This area was a typical river-dominated ocean margin (RiOMar) based on the Cao et al. framework [1]. The RiOMar is mostly comprised of shelf regions featuring major nutrient loadings from riverine input, including the far-reaching area of the river plume. In this study, *Trichodesmium* had the highest abundance, followed by γ-24774A11 and UCYN-A. Moreover, qPCR quantities of *Trichodesmium* were abundant in the site controlled by the runoff. The literature also revealed, using microscopic examination, that *Trichodesmium* was abundant in a similar habitat [56]. Occurrences of *Trichodesmium* dominance were also revealed in the Amazon plume-influenced regions [21,57]. Inversely, qPCR quantities of Proteobacterial γ-24774A11 were more abundant away from the coast where the riverine discharge fades. With the exception of *Trichodesmium*, unicellular cyanobacteria and Proteobacteria were at low abundance levels in the river-dominated SCS. Furthermore, the relative contribution of Cyanobacteria (OTUs) and the genic abundance of *Trichodesmium* decreased with offshore distance in the neritic area, in contrast to the relative contribution of non-cyanobacteria and abundance of the main Proteobacterial diazotrophs. Our results were comparable to those of Kong et al. [30]. Kong et al. suggested that γ-Proteobacteria formed the dominant diazotrophic group, with cyanobacterial diazotrophs accounting for only a relatively small proportion in the oceanic waters of the northern South China Sea [30]. Consistently, similar patterns of diazotrophic communities were revealed in the Mekong River plume and the Amazon River plume [5,58]. The highly heterogeneous diazotrophic community in this region influenced by terrestrial input was presumably caused by the remarkably different responses of different diazotrophic groups to the steep environmental gradient [59]. It is worth noting that environmental conditions other than ammonium had no significant influence on the composition of the diazotrophic community in this study. The important influence of ammonium on the diazotrophic community was also observed in the Eastern Indian Ocean (EIO) [24]. Farnelid et al. also found that genes and transcripts of diazotrophs were abundant in ammonium-rich waters [60]. In addition, the unique site Stn.18 was located on the continental shelf near Hainan Island, where it is frequently affected by coastal upwellings [61]. Zhang et al. (2015) revealed that the surface density of *Trichodesmium* reached 2797 trichomes L^−1^ [62]. Assuming that one typical trichome consists of approximately 100 cells [63] and that each cell contains one genome [33], the abundance of *Trichodesmium* gene copies in our study was estimated to have been 2844 trichomes L^−1^, which is comparable to the findings of Zhang et al. [62].

In the area in the western boundary of the sSCS, the Vietnamese upwelling acts as one of the most important pumps that push the nutrient-rich deep water into the euphotic zone in the wSCS region [64,65]. In contrast to the northern sSCS, the structure of the diazotrophic community was different in the western boundary of the SCS. The proportion of Proteobacteria in the community was compared to that of Cyanobacteria in this region. Specifically, the abundance of *Trichodesmium* was comparable to that of Proteobacteria γ-24774A11. Low abundances of unicellular cyanobacterial groups UCYN-A, *Crocosphaera watsonii*, and UCYN-C were observed in wSCS, and UCYN-C was detected sporadically. Genus γ-Proteobacteria was shown to be thriving in the thermohaline site, which is consistent with the positive relationship between γ-Proteobacteria and temperature. The regional variation in diazotrophic community composition provides evidence of ecological niche partitioning. The maximum abundances of *nifH* gene copies affiliated with *Trichodesmium* in our study were consistent with those reported by Moisander et al. [33]; however, the maximum abundances of *nifH* gene copies affiliated to UCYN-A, *Crocosphaera watsonii*, and α-Proteobacteria were one order of magnitude higher in our study compared with the numbers reported by Moisander et al. [33]. Moreover, the abundances of *nifH* gene copies affiliated with γ-Proteobacteria, an abundant population in our study, was two orders of magnitude higher in our study than that in Moisander et al. [33]. Conversely, Zhang et al. revealed that the *nifH* copies of the γ-Proteobacteria group were at a relatively low abundance, and the maximum abundance reported was one order of magnitude lower than that in our study [32].

The compositional variation of the diazotrophic communities demonstrated their potential contribution to the local habitat. In the RiOMar, which has major nutrient loadings from the Pearl River input, the genetic abundance of *Trichodesmium* dominated the diazotrophic community in the river plume. Although sufficient nutrients promoted *Trichodesmium* thriving, Wu et al. (2018) calculated that the N_2_ fixation rate of *Trichodesmium* in this region was not higher than other regions as expected [66]. This phenomenon potentially provided a foil for the biological pump efficacy of *Trichodesmium* as a phytoplankton lineage. The process of *Trichodesmium* bloom greatly elevated excretions of transparent exopolymer particles (TEP) and a massive downward pulse of particulate organic matter (POM), thereby facilitating vertical fluxes of carbon and nitrogen [66]. Weber et al. found that *Trichodesmium* made much greater contributions to the particulate nitrogen pool and to CO_2_ drawdown compared to other diazotrophs in the Amazon plume [67]. Rees et al.’s observation of metabolically active *Trichodesmium* suggested the potential for viable and potentially active *Trichodesmium* carbon fixation rather than N_2_ fixation in coastal and nutrient-depleted water [68]. It is speculated that *Trichodesmium* contributed significantly to oceanic carbon and nitrogen cycling through this pathway in the survey area. Heterotrophic diazotrophs occupied a potential niche in the ecosystem. Inomura et al. (2018) determined that N_2_ fixation by the heterotrophic diazotroph occurred in the condition of imposing ammonium concentration using a metabolic model, and the modeling result was consistent with the laboratory data [69]. The significant relation between diazotrophs and ammonium in this study provided evidence for understanding the ecological advantage of heterotrophic diazotrophs.

In contrast, the western boundary of the SCS was subjected to significant N-limitation, although the upwelling region off Vietnam drove nutrients to the surface water from below the thermocline. Diazotroph activities represented a critical external nitrogen input to seawaters, supplying available nitrogen for biotic growth [5]. Field observations in the Vietnamese upwelling have verified that N_2_ fixation by diazotrophs was an important new nitrogen source for the local ecosystem [64]. Subramaniam et al. (2013) found that nitrogen fixation rates were two to seven times higher in the upwelling region than in non-upwelling periods in the equatorial Atlantic [70], suggesting the importance of diazotroph activities in upwelling regions. In our results, the relative contribution of Proteobacteria was comparable to that of Cyanobacteria in the community, suggesting that Proteobacteria and Cyanobacteria were probably given equal roles in the western boundary of the SCS. 

### 4.2. Significance of Diazotrophs in the Oligotrophic Oceanic SCS

The cSCS survey covered a vast basin that is often driven by a large-scale cyclonic gyre, which closely follows the alternating monsoons. Most sampling stations (excluding Stn.18) of cSCS were in the oligotrophic oceanic area, with water depths of 2000–4400 m. The cSCS could be identified as an ocean-dominated margin (OceMar) system, wherein nutrients are sourced from the open ocean [1]. In this OceMar system, diazotrophs belonging to the phyla Proteobacteria became abundant, followed by *Trichodesmium*. In particular, α-Proteobacteria (*Sagittula castanea*) was prevalent in the basin. In comparison to RiOMar, the abundance of Proteobacterial diazotrophs increased while *Trichodesmium* decreased in the OceMar, whereas the abundance of unicellular cyanobacteria groups in the OceMar was comparable to those in RiOMar. Chen et al. also determined that Proteobacteria accounted for 77% of the overall *nifH* clone library, of which α-Proteobacterial phylotypes accounted for almost 80% of the Proteobacteria [34], thereby, demonstrating their relative importance in the diazotrophic community in the OceMar system. Comparably, a diverse diazotrophic community, in particular, abundant α-Proteobacteria and γ-Proteobacteria, was present in the open ocean Arabian Sea [71,72]. The prevalence of α-Proteobacteria and γ-Proteobacteria reflect their ecological importance in the OceMar ecosystem, particularly under conditions where cyanobacterial diazotrophs were not dominant. In the cSCS, the frequent mesoscale eddies affect nutrient supply to the euphotic zone [73], the seasonal intrusion of the Kuroshio from the Pacific Ocean changes the nutrient stock in the euphotic waters as well [40]. There were obvious differences in the nutrient concentrations in the cSCS region, among which phosphate concentrations were much lower compared with those in other regions. Consequently, the N/P ratio was relatively high in the cSCS, which was approximately twice that of the Redfield ratio (N/P ratio 16/1) in the typical site (Stn.12). Notably, a significant positive relationship was revealed between the relative abundance of Proteobacteria and nitrite concentration, particularly gene copies of *Sagittula castanea*, the maximum abundance (2.25 × 10^7^ copies L^−1^) occurred at the site (Stn.12), which had the maximum N/P ratio. *Sagittula castanea* (α-Proteobacteria) and γ-24774A11 (γ-Proteobacteria) were most abundant in the cSCS during our study. *Sagittula castanea* was found to be more dominating in the equatorial region and offshores in the Eastern Indian Ocean [24]. Besides, a more homogeneous distribution of γ-24774A11 agreed well with a previous study conducted in the South Pacific Ocean [22,74]. In addition, the highest abundance of *Trichodesmium* occurred in the northern boundary area controlled by the Kuroshio intrusion (KI) during the cruise. This result was comparable to Lu et al.’s observation that the KI exhibits a significantly high abundance of diazotrophs [31]. These results suggested that the biogeographic distribution of diazotrophs was modulated by close associations between diazotrophs and the KI. Moreover, a significant negative correlation between the relative abundance of Cyanobacteria and temperature was detected in this study. Chen et al. found the *nifH* gene abundance of *Sagittula castanea* had a significant negative correlation with temperature [36].

The superiority of α-Proteobacteria and γ-Proteobacteria reflected their significance as major fixers in the OceMar ecosystem. Taking the taxa *Sagittula castanea* as an example, assume that the N_2_ fixation rate of *Sagittula castanea* was 0.06 fmol N cell^−1^ d^−1^, which is the maximum cell-specific N_2_ fixation rate of the isolated *Sagittula castanea* strain P11 [75]. The approximate estimate of *Sagittula castanea* N_2_ fixation rate was potentially up to 5.01 nmol N L^−1^ d^−1^ throughout the whole SCS. This rate partly exceeded the *Trichodesmium* N_2_ fixation rate in the northern SCS [15], as well as the rates of the cyanobacterial-dominated community in the western SCS [58]. Our results suggested that the activity of Proteobacterial diazotrophs significantly contributed to the nitrogen budget, particularly when cyanobacterial diazotrophs were not superior. Moreover, a mathematical model provided a testable hypothesis that unicellular heterotrophic bacteria growing on sinking marine particles could fix N_2_ under suitable environmental conditions [76], suggesting that heterotrophic diazotrophs played important roles both in oceanic nitrogen cycling and carbon sequestration.

### 4.3. Potential Contribution of Diazotrophs in the Pelagic WPO

The complex hydrological conditions of WBCs have an evident impact on the variability of diazotroph assemblages [77]. Compared with the SCS, the thriving population of *Crocosphaera watsonii* (UCYN-B) was a remarkable feature in the pelagic WPO, the gene abundance of which was two orders of magnitude higher than that in the SCS. With the exception of *Trichodesmium* and *Crocosphaera watsonii*, the abundances of Proteobacterial diazotrophs, UCYN-A and UCYN-C, were equal to those in the SCS. In the WPO, the abundance of *Trichodesmium*, as well as that of *Crocosphaera watsonii* and UCYN-C, were relatively high in the north edge of the WPO survey field that is controlled by the upstream Kuroshio, which was comparable to that in the SCS influenced by the Kuroshio intrusion. This pattern is consistent with previous studies [15,35], indicating that the oceanic conditions in the two regions are favorable for the growth of unicellular and filamentous diazotrophs. Notably, symbiont *Richelia intracellularis* was detected in this study; however, the contribution of *Richelia intracellularis* to the diazotrophic community was very limited. Shiozaki et al.’s (2018) results indicated that *Richelia* associated with *Rhizosolenia* or *Hemiaulus* were common in the surface water of the Kuroshio [78]. In the regions north of 5° N, our results showed that abundances of UCYN-B and Proteobacterial diazotrophs increased concomitantly with the reduction of *Trichodesmium* abundance, indicating a replacement of the dominance of *Trichodesmium*. The prevalent gene expression of γ-24774A11 and UCYN-B were also reported in the literature [13,36]. Abundant *Sagittula castanea* that was infrequent in previous investigations was observed in this study. Furthermore, relatively low abundances of UCYN-A and UCYN-C varied negatively with temperature, which together with γ-24774A11, had significant positive correlations with salinity in the WPO. Our results provide evidence for the adaptation of unicellular cyanobacteria and γ-Proteobacteria diazotroph to low temperatures and high salinity in the western Pacific [22,79]. 

In contrast, in the subequatorial zone south of 5° N, situated in the western Pacific warm pool, extremely high abundances of *Trichodesmium* were recorded in the N-depleted aquatic environment (an N/P ratio of 122.62), potentially as a result of beneficial effects of the Mindanao Current (HE) curl and the North Equatorial Counter Current (NECC). This phenomenon indicated that *Trichodesmium* readily blooms in this region. This zone is usually regarded as a hotspot of N_2_ fixation, attributed to the occurrence of *Trichodesmium* [13]. It was observed that planktonic cyanobacterial diazotrophs thrive concomitantly with N_2_ fixation activities in the WPWP, and primary production was significantly correlated with N_2_ fixation rather than nitrate assimilation, indicating that N_2_ fixation-derived nitrogen was the main source of primary production, as well as the greatest driver of change to the nutrient inventory [80]. Moreover, Shiozaki et al. (2017) observed that significantly low N_2_ fixation was rarely observed in the case of *Trichodesmium*, and the N_2_ fixation hot spot was attributed to the occurrence of *Trichodesmium* [13]. The significant amount of *Trichodesmium* found in this study indicates the potential importance of this N_2_ fixation group in the WPO. Furthermore, according to the literature, diazotrophs thriving concomitantly with N_2_ fixation activities have been observed in the WPWP, and the contribution of diazotrophs to changing the nutrient inventory in the pelagic WPO exceeded that in the SCS and the upstream Kuroshio, as well as that in the North Pacific Subtropical Gyre [12,13,35,66]. *Trichodesmium*, as the dominant photosynthetic diazotroph in the surface ocean, seemingly carried out the two conflicting metabolic processes of N_2_ fixation and photosynthesis. Inomura et al. (2019) suggested that respiratory protection, trichome formation, and diffusion barriers represented essential strategies of *Trichodesmium*. These factors facilitated the growth of *Trichodesmium* and permitted them to become a major source of new nitrogen in the oligotrophic aerobic ocean [81].

## 5. Conclusions

Diazotroph activities in the surface ocean profoundly influence the global marine nitrogen cycle and primary productivity. In this study, Proteobacteria combined with Cyanobacteria dominated the diazotrophic community in the SCS and Cyanobacteria dominated in the diazotrophic community in the WPO. Our results highlight the need to comprehensively understand and model biogeochemical cycles on local and global scales. A limitation of this study is the lack of micronutrient analyses, such as that of iron. We also did not conduct an in situ nitrogen fixation rate assay. Our results provided potential evidence for comprehensively understanding and modeling biochemical cycles on local and global scales.

## Figures and Tables

**Table 1:** Primers, Taqman probes, and standard clones for quantitative polymerase chain reaction (qPCR) analysis targeting *nifH* gene of different cyanobacterial diazotrophic groups. The 5′ and 3′ of TaqMan probes were labeled with the fluorescent reporter FAM (6-carboxyfluorescein) and the quenching dye TAMRA (6-carboxytetramethylrhodamine), respectively.

Targets	Forward Primer (5′–3′)	Probe	Reverse Primer (5′–3′)	Reference
*Trichodesmium*	GACGAAGTATTGAAGCCAGGTTTC	CATTAAGTGTGTTGAATCTGGTGGTCCTGAGC	ACGGCCAGCGCAACCTA	21
UCYN-A	GGTTACAACAACGTTTTATGTGTTGA	TCTGGTGGTCCTGAGCCCGGA	GCAGTAATAATACCACGACCAGCAC	21
UCYN-B	TGGTCCTGAGCCTGGAGTTG	TGTGCTGGTCGTGGTAT	CTTCTTCTAGGAAGTTGATGGAGGTG	21
UCYN-C	ATACCAAGGAATCAAGTGTGTTGAGT	CGGTGGTCCCGAGCCTGGAG	ATACCAAGGAATCAAGTGTGTTGAGT	21
γ-24774A11	TCCACACGTCTTATTCTGCACT	AAGTGCTTAAGGTTGGCTTTGGCGACA	AGAGCAAACAATGTAGATTTCCTG	22
*Sagittula castanea*	ATCACCGCCATCAACTTCCT	CGCCTACGATGACGTGGATTACGTGTCC	AGACCACGTCGCCCAGAAC	32

## Data Availability

The data presented in this study are openly available in the NCBI Sequence Read Achieve database (https://submit.ncbi.nlm.nih.gov/subs/sra/SUB7406573/overview, accessed on 13 May 2020).

## Supplementary Materials

The following are available online at https://www.mdpi.com/article/10.3390/biology10060555/s1, Table S1: The principal coordinates of neighbor matrices (PCNM) analysis based on geographic coordinates.

## References

[1] Cao Z.M., Yang W., Zhao Y.Y., Guo X.H., Yin Z.Q., Du C.J., Zhao H.D., Dai M.H. (2019). Diagnosis of CO_2_ dynamics and fluxes in global coastal oceans. Natl. Sci. Rev..

[2] Yang H.J., Liu Q.Y., Liu Z.Y., Wang D.X., Liu X.B. (2002). A general circulation model study of the dynamics of the upper ocean circulation of the South China Sea. J. Geophys. Res. Ocean..

[3] Guo M.X., Chai F., Xiu P., Li S.Y., Rao S. (2015). Impacts of mesoscale eddies in the South China Sea on biogeochemical cycles. Ocean Dynam..

[4] Cai W.J., Dai M.H., Wang Y.C., Zhai W.D., Huang T., Chen S.T., Zhang F., Chen Z.Z., Wang Z.H. (2004). The biogeochemistry of inorganic carbon and nutrients in the Pearl River estuary and the adjacent Northern South China Sea. Cont. Shelf. Res..

[5] Voss M., Bombar D., Loick N., Dippner J.W. (2006). Riverine influence on nitrogen fixation in the upwelling region off Vietnam, South China Sea. Geophys. Res. Lett..

[6] Yang Q.X., Zhou L., Tian J.W., Zhao W. (2013). The Roles of Kuroshio Intrusion and Mesoscale Eddy in Upper Mixing in the Northern South China Sea. J. Coast. Res..

[7] Hu D., Wu L., Cai W., Gupta A.S., Ganachaud A., Qiu B., Gordon A.L., Lin X., Chen Z., Hu S. (2015). Pacific western boundary currents and their roles in climate. Nature.

[8] Moore C.M., Mills M.M., Arrigo K.R., Berman-Frank I., Bopp L., Boyd P.W., Galbraith E.D., Geider R.J., Guieu C., Jaccard S.L. (2013). Processes and patterns of oceanic nutrient limitation. Nat. Geosci..

[9] Wu J.F., Chung S.W., Wen L.S., Liu K.K., Chen Y.L.L., Chen H.Y., Karl D.M. (2003). Dissolved inorganic phosphorus, dissolved iron, and *Trichodesmium* in the oligotrophic South China Sea. Glob. Biogeochem. Cycles.

[10] Karl D., Letelier R., Tupas L., Dore J., Christian J., Hebel D. (1997). The role of nitrogen fixation in biogeochemical cycling in the subtropical North Pacific Ocean. Nature.

[11] Zehr J.P., Capone D.G. (2020). Changing perspectives in marine nitrogen fixation. Science.

[12] Sohm J.A., Subramaniam A., Gunderson T.E., Carpenter E.J., Capone D.G. (2011). Nitrogen fixation by *Trichodesmium* spp. and unicellular diazotrophs in the North Pacific Subtropical Gyre. J. Geophys. Res..

[13] Shiozaki T., Bombar D., Riemann L., Hashihama F., Takeda S., Yamaguchi T., Ehama M., Hamasaki K., Furuya K. (2017). Basin scale variability of active diazotrophs and nitrogen fixation in the North Pacific, from the tropics to the subarctic Bering Sea. Glob. Biogeochem. Cycles.

[14] Chen L.Y.L., Chen H.Y., Lin Y.H. (2003). Distribution and downward flux of *Trichodesmium* in the South China Sea as influenced by the transport from the Kuroshio Curren. Mar. Ecol. Prog. Ser..

[15] Chen L.Y.L., Chen H.Y., Tuo S.H., Ohki K. (2008). Seasonal dynamics of new production from *Trichodesmium* N_2_ fixation and nitrate uptake in the upstream Kuroshio and South China Sea basin. Limnol. Oceanogr..

[16] Tuo S.H., Chen Y.L.L., Chen H.Y. (2014). Low nitrate availability promotes diatom diazotroph associations in the marginal seas of the western Pacific. Aquat. Microb. Ecol..

[17] Farnelid H., Andersson A.F., Bertilsson S., Al-Soud W.A., Hansen L.H., Sorensen S., Steward G.F., Hagstrom A., Riemann L. (2011). Nitrogenase gene amplicons from global marine surface waters are dominated by genes of non-cyanobacteria. PLoS ONE.

[18] Zehr J.P., Mellon M.T., Zani S. (1998). New Nitrogen-Fixing Microorganisms Detected in Oligotrophic Oceans by Amplification of Nitrogenase (*nifH*) Genes. Appl. Environ. Microbiol..

[19] Cui L., Yang K., Li H., Zhang H., Su J., Paraskevaidi M., Martin F., Ren B., Zhu Y. (2018). Functional single-cell approach to probing nitrogen-fixing bacteria in soil communities by resonance Raman spectroscopy with ^15^N_2_ labeling. Anal. Chem..

[20] Bonnet S., Caffin M., Berthelot H., Grosso O., Benavides M., Helias-Nunige S., Guieu C., Stenegren M., Foster R.A. (2018). In-depth characterization of diazotroph activity across the western tropical South Pacific hotspot of N_2_ fixation (OUTPACE cruise). Biogeosciences.

[21] Goebel N.L., Turk K.A., Achilles K.M., Paerl R., Hewson I., Morrison A.E., Montoya J.P., Edwards C.A., Zehr J.P. (2010). Abundance and distribution of major groups of diazotrophic cyanobacteria and their potential contribution to N_2_ fixation in the tropical Atlantic Ocean. Environ. Microbiol..

[22] Moisander P.H., Beinart R.A., Hewson I., White A.E., Johnson K.S., Carlson C.A., Montoya J.P., Zehr J.P. (2010). Unicellular cyanobacterial distributions broaden the oceanic N_2_ fixation domain. Science.

[23] Stenegren M., Caputo A., Berg C., Bonnet S., Foster R.A. (2018). Distribution and drivers of symbiotic and free-living diazotrophic cyanobacteria in the western tropical South Pacific. Biogeosciences.

[24] Wu C., Kan J., Liu H., Pujari L., Guo C., Wang X., Sun J. (2019). Heterotrophic Bacteria Dominate the Diazotrophic Community in the Eastern Indian Ocean (EIO) during Pre-Southwest Monsoon. Microb. Ecol..

[25] Zehr J.P., Jenkins B.D., Short S.M., Steward G.F. (2003). Nitrogenase gene diversity and microbial community structure: A cross-system comparison. Environ. Microbiol..

[26] Luo Y.W., Doney S.C., Anderson L.A., Benavides M., Berman-Frank I., Bode A., Bonnet S., Bostrom K.H., Bottjer D., Capone D.G. (2012). Database of diazotrophs in global ocean: Abundance, biomass and nitrogen fixation rates. Earth Syst. Sci. Data.

[27] Xiao P., Jiang Y.G., Liu Y., Tan W.H., Li W.H., Li R.H. (2015). Re-Evaluation of the diversity and distribution of diazotrophs in the South China Sea by pyrosequencing the *nifH* gene. Mar. Freshw. Res..

[28] Grosse J., Bombar D., Hai N.D., Lam N.N., Voss M. (2010). The Mekong River plume fuels nitrogen fixation and determines phytoplankton species distribution in the South China Sea during low- and high-discharge season. Limnol. Oceanogr..

[29] Yang Q.S., Dong J.D., Zhang Y.Y., Ling J., Wang D.X., Wu M.L., Jiang Y.F., Zhang Y.Z. (2015). Diversity analysis of diazotrophs associated with corals from Xisha and Sanya, South China Sea. Aquat. Ecosyst. Health.

[30] Kong L.L., Jing H.M., Kataoka T., Sun J., Liu H.B. (2011). Phylogenetic diversity and spatio-temporal distribution of nitrogenase genes (*nifH*) in the northern South China Sea. Aquat. Microb. Ecol..

[31] Lu Y.Y., Wen Z.Z., Shi D.L., Lin W.F., Bonnet S., Dai M.H., Kao S.J. (2019). Biogeography of N_2_ Fixation Influenced by the Western Boundary Current Intrusion in the South China Sea. J. Geophys. Res. Oceans.

[32] Zhang Y., Zhao Z., Sun J., Jiao N. (2011). Diversity and distribution of diazotrophic communities in the South China Sea deep basin with mesoscale cyclonic eddy perturbations. FEMS Microbiol. Ecol..

[33] Moisander P.H., Beinart R.A., Voss M., Zehr J.P. (2008). Diversity and abundance of diazotrophic microorganisms in the South China Sea during intermonsoon. ISME J..

[34] Chen T.Y., Chen L.Y.L., Sheu D., Chen H.Y., Lin Y.H., Shiozaki T. (2019). Community and abundance of heterotrophic diazotrophs in the northern South China Sea: Revealing the potential importance of a new alphaproteobacterium in N_2_ fixation. Deep Sea Res. Part I Oceanogr. Res. Pap..

[35] Chen Y.L., Chen H.Y., Lin Y.H., Yong T.C., Taniuchi Y., Tuo S.H. (2014). The relative contributions of unicellular and filamentous diazotrophs to N_2_ fixation in the South China Sea and the upstream Kuroshio. Deep Sea Res. Part I Oceanogr. Res. Pap..

[36] Chen M.M., Lu Y.Y., Jiao N.Z., Tian J.W., Kao S.J., Zhang Y. (2019). Biogeographic drivers of diazotrophs in the Western Pacific Ocean. Limnol. Oceanogr..

[37] Zehr J.P. (2011). Nitrogen fixation by marine cyanobacteria. Trends Microbiol..

[38] Bombar D., Paerl R.W., Riemann L. (2016). Marine Non-cyanobacterial diazotrophs: Moving beyond molecular detection. Trends Microbiol..

[39] Dippner J.W., Nguyen K.V., Hein H., Ohde T., Loick N. (2006). Monsoon-induced upwelling off the Vietnamese coast. Ocean Dynam..

[40] Du C., Liu Z., Dai M., Kao S.J., Cao Z., Zhang Y., Huang T., Wang L., Li Y. (2013). Impact of the Kuroshio intrusion on the nutrient inventory in the upper northern South China Sea: Insights from an isopycnal mixing model. Biogeosciences.

[41] Grasshoff K., Kremling K., Ehrhardt M. (2009). Methods of Seawater Analysis.

[42] Caporaso J.G., Kuczynski J., Stombaugh J., Bittinger K., Bushman F.D., Costello E.K., Fierer N., Pena A.G., Goodrich J.K., Gordon J.I. (2010). QIIME allows analysis of high-throughput community sequencing data. Nat. Methods.

[43] Magoc T., Salzberg S.L. (2011). FLASH: Fast length adjustment of short reads to improve genome assemblies. Bioinformatics.

[44] Kunin V., Engelbrektson A., Ochman H., Hugenholtz P. (2010). Wrinkles in the rare biosphere: Pyrosequencing errors can lead to artificial inflation of diversity estimates. Environ. Microbiol..

[45] Edgar R.C. (2010). Search and clustering orders of magnitude faster than BLAST. Bioinformatics.

[46] Altschul S.F., Madden T.L., Schaffer A.A., Zhang J., Zhang Z., Miller W., Lipman D.J. (1997). Gapped BLAST and PSI-BLAST: A new generation of protein database search programs. Nucleic Acids Res..

[47] Sudhir K., Glen S., Michael L., Christina K., Koichiro T. (2018). Mega X: Molecular evolutionary genetics analysis across computing platforms. Mol. Biol. Evol..

[48] Letunic I., Bork P. (2019). Interactive Tree of Life (iTOL) v4: Recent updates and new developments. Nucleic Acids Res..

[49] Eric G., Graybill V. (2014). R Development Core Team (2008). R: A language and Environment for Statistical Computing.

[50] RCRTeam (2019). R: A Language and Environment for Statistical Computing, Version 3.6.1. Vienna, Austria..

[51] Schlitzer R. Ocean Data View. https://odv.awi.de.

[52] Oksanen J., Blanchet F.G., Kindt R., Legendre P., Minchin P.R., O’Hara R.B., Simpson G.L., Solymos P., Stevens M.H.H., Wagne R.H. Vegan: Community Ecology Package. R. Package Version p. 2.0-9.

[53] Wang Z.B., Sun Y.Y., Li Y., Chen X.L., Wang P., Ding H.T., Chen B., Zhang X.Y., Song X.Y., Wang M. (2020). Significant Bacterial Distance-Decay Relationship in Continuous, Well-Connected Southern Ocean Surface Water. Microb. Ecol..

[54] Dray S., Legendre P., Peres-Neto P.R. (2006). Spatial modelling: A comprehensive framework for principal coordinate analysis of neighbour matrices (PCNM). Ecol. Model..

[55] McMurdie P.J., Holmes S. (2013). Phyloseq: An R package for reproducible interactive analysis and graphics of microbiome census data. PLoS ONE.

[56] Xue B., Sun J., Li T.T. (2016). Phytoplankton community structure of northern South China Sea in summer of 2014. Haiyangxuebao.

[57] Weber S.C., Carpenter E.J., Coles V.J., Yager P.L., Goes J., Montoya J.P. (2017). Amazon River influence on nitrogen fixation and export production in the western tropical North Atlantic. Limnol. Oceanogr..

[58] Bombar D., Moisander P.H., Dippner J.W., Foster R.A., Voss M., Karfeld B., Zehr J.P. (2011). Distribution of diazotrophic microorganisms and *nifH* gene expression in the Mekong River plume during intermonsoon. Mar. Ecol. Prog. Ser..

[59] Cheung S., Suzuki K., Saito H., Umezawa Y., Xia X., Liu H. (2017). Highly heterogeneous diazotroph communities in the Kuroshio Current and the Tokara Strait, Japan. PLoS ONE.

[60] Farnelid H., Bentzon-Tilia M., Andersson A.F., Bertilsson S., Jost G., Labrenz M., Jurgens K., Riemann L. (2013). Active nitrogen-fixing heterotrophic bacteria at and below the chemocline of the central Baltic Sea. ISME J..

[61] Jing Z.Y., Qi Y.Q., Du Y. (2011). Upwelling in the continental shelf of northern South China Sea associated with 1997–1998 El Niño. J. Geophys. Res. Oceans.

[62] Zhang R., Chen M., Yang Q., Lin Y.S., Mao H.B., Qiu Y.S., Tong J.L., Lv E., Yang Z., Yang W.F. (2015). Physical-Biological coupling of N_2_ fixation in the northwestern South China Sea coastal upwelling during summer. Limnol. Oceanogr..

[63] Capone D.G., Zehr J.P., Paerl H.W., Bergman B., Carpenter E.J. (1997). *Trochodesmium*, a Globally Significant Marine Cyanobacterium. Science.

[64] Bombar D., Dippner J.W., Hai N.D., Lam N.N., Liskow I., Loick-Wilde N., Voss M. (2010). Sources of new nitrogen in the Vietnamese upwelling region of the South China Sea. J. Geophys. Res. Oceans.

[65] Loick N., Dippner J., Doan H.N., Liskow I., Voss M. (2007). Pelagic nitrogen dynamics in the Vietnamese upwelling area according to stable nitrogen and carbon isotope data. Deep Sea Res. Pt. I.

[66] Wu C., Fu F.X., Sun J., Thangaraj S., Pujari L. (2018). Nitrogen fixation by *Trichodesmium* and unicellular diazotrophs in the northern South China Sea and the Kuroshio in summer. Sci. Rep..

[67] Bar-Zeev E., Avishay I., Bidle K.D., Berman-Frank I. (2013). Programmed cell death in the marine cyanobacterium *Trichodesmium* mediates carbon and nitrogen export. ISME J..

[68] Rees A.P., Tait K., Widdicombe C.E., Quartly G.D., McEvoy A.J., Al-Moosawi L. (2016). Metabolically active, non-nitrogen fixing, *Trichodesmium* in UK coastal waters during winter. J. Plankton Res..

[69] Inomura K., Bragg J., Riemann L., Follows M.J. (2018). A quantitative model of nitrogen fixation in the presence of ammonium. PLoS ONE.

[70] Subramaniam A., Mahaffey C., Johns W., Mahowald N. (2013). Equatorial upwelling enhances nitrogen fixation in the Atlantic Ocean. Geophys. Res. Lett..

[71] Bird C., Wyman M. (2013). Transcriptionally active heterotrophic diazotrophs are widespread in the upper water column of the Arabian Sea. FEMS Microbiol. Ecol..

[72] Jayakumar A., Al-Rshaidat M.M., Ward B.B., Mulholland M.R. (2012). Diversity, distribution, and expression of diazotroph *nifH* genes in oxygen-deficient waters of the Arabian Sea. FEMS Microbiol. Ecol..

[73] Xiu P., Chai F. (2011). Modeled biogeochemical responses to mesoscale eddies in the South China Sea. J. Geophys. Res. Oceans.

[74] Moisander P.H., Serros T., Paerl R.W., Beinart R.A., Zehr J.P. (2014). GammaProteobacterial diazotrophs and *nifH* gene expression in surface waters of the South Pacific Ocean. ISME J..

[75] Martínez-Pérez C., Mohr W., Schwedt A., Dürschlag J., Callbeck C.M., Schunck H., Dekaezemacker J., Buckner C.R.T., Lavik G., Fuchs B.M. (2018). Metabolic versatility of a novel N_2_ -fixing *Alphaproteobacterium* isolated from a marine oxygen minimum zone. Environ. Microbiol..

[76] Chakraborty S., Andersen K., Visser A., Inomura K., Follows M., Riemann L. (2020). Quantifying nitrogen fixation by heterotrophic bacteria in sinking marine particles. Nat. Commun..

[77] Church M.J., Mahaffey C., Letelier R.M., Lukas R., Zehr J.P., Karl D.M. (2009). Physical forcing of nitrogen fixation and diazotrophic community structure in the North Pacific subtropical gyre. Glob. Biogeochem. Cycles.

[78] Shiozaki T., Kondo Y., Yuasa D., Takeda S. (2018). Distribution of major diazotrophs in the surface water of the Kuroshio from northeastern Taiwan to south of mainland Japan. J. Plankton Res..

[79] Bonnet S., Rodier M., Turk-Kubo K.A., Germineaud C., Menkes C., Ganachaud A., Cravatte S., Raimbault P., Campbell E., Quéroué F. (2015). Contrasted geographical distribution of N_2_ fixation rates and *nifH* phylotypes in the Coral and Solomon Seas (southwestern Pacific) during austral winter conditions. Glob. Biogeochem. Cycles.

[80] Shiozaki T., Kodama T., Kitajima S., Sato M., Furuya K. (2013). Advective transport of diazotrophs and importance of their nitrogen fixation on new and primary production in the western Pacific warm pool. Limnol. Oceanogr..

[81] Inomura K., Wilson S.T., Deutsch C. (2019). Mechanistic model for the coexistence of nitrogen fixation and photosynthesis in marine *Trichodesmium*. Systems.

