# Supplementary material for: Comparison of Diazotrophic Composition and Distribution in the South China Sea and the Western Pacific Ocean
Source: Biology (Basel). 2021 Jun 20;10(6):555. doi: 10.3390/biology10060555 (PMC8235572; doi:10.3390/biology10060555)
Supplement: Supplementary file 1 [file biology-10-00555-s001.zip › biology-1251643-supplementary.pdf]

Table S1. The principal coordinates of neighbor matrices (PCNM) analysis based on geographic coordinates.

Station	PCNM1	PCNM2	PCNM3	PCNM4	PCNM5	PCNM6	PCNM7	PCNM8	PCNM9	PCNM10	PCNM11	PCNM12	PCNM13	PCNM14
1	-0.1037	-0.2850	-0.1176	0.3421	-0.0982	0.0262	0.3245	-0.1814	-0.0406	-0.0575	0.0231	0.5345	0.0970	-0.0141
2	-0.1138	-0.2474	-0.1250	0.0933	0.1744	-0.0320	-0.1312	0.0638	-0.0315	-0.2014	0.2305	-0.2190	-0.0398	0.0060
3	-0.0469	0.4213	0.2196	0.2507	0.1750	-0.4470	0.0806	0.0147	0.0124	0.0105	-0.0245	0.0001	0.0000	0.0000
4	-0.1146	-0.2486	-0.1258	0.0824	0.1707	-0.0348	-0.1341	0.0666	-0.0563	-0.0375	0.0100	-0.1850	-0.0327	0.0032
5	-0.1325	0.0173	0.0189	0.0758	-0.0081	-0.0658	0.0487	-0.0046	0.0063	-0.6926	-0.2175	-0.1033	-0.0240	0.0138
6	-0.1235	-0.1764	-0.0863	-0.1438	-0.0912	-0.0201	0.0203	0.0214	0.6415	0.0468	-0.0239	-0.0003	-0.0001	0.0000
7	-0.0704	0.3857	0.2036	0.0596	-0.0721	0.4010	-0.0591	-0.0288	-0.3744	-0.0547	0.0039	-0.0002	0.0000	0.0000
8	-0.0923	0.2980	0.1581	-0.3361	-0.3369	0.0136	0.1353	-0.0302	0.3768	0.0035	-0.0104	0.0004	0.0001	0.0000
9	-0.1334	0.0483	0.0339	0.0429	-0.0208	-0.0715	0.0368	0.0083	-0.0493	0.1739	0.4815	0.0084	-0.0008	0.0047
10	-0.1337	0.0454	0.0329	0.0590	-0.0159	-0.0702	0.0419	0.0049	-0.0205	-0.1185	0.5336	-0.0361	-0.0066	0.0014
11	-0.1308	-0.0811	-0.0350	-0.1284	-0.1555	0.4013	-0.0561	-0.0165	-0.0850	-0.2103	-0.0319	-0.0280	-0.0020	-0.0055
12	-0.1294	-0.0735	-0.0317	-0.1486	-0.1614	0.3994	-0.0622	-0.0132	-0.1198	0.1960	0.0384	0.0263	0.0017	0.0055
13	-0.1355	0.0292	0.0240	0.0351	-0.0228	-0.0708	0.0343	0.0101	-0.0682	0.1048	-0.2197	0.0257	0.0072	-0.0055
14	-0.1334	0.0308	0.0243	0.0182	-0.0269	-0.0714	0.0296	0.0109	-0.0974	0.3921	-0.3086	0.0649	0.0056	0.0109
15	-0.1127	-0.1903	-0.0975	-0.4335	-0.2428	-0.3479	0.1495	-0.0239	-0.2264	-0.0435	0.0314	-0.0002	0.0000	0.0000
16	-0.1356	0.0225	0.0209	0.0450	-0.0193	-0.0695	0.0376	0.0072	-0.0514	-0.1256	-0.3464	-0.0047	0.0023	-0.0061
17	-0.1353	0.0384	0.0288	0.0400	-0.0219	-0.0713	0.0356	0.0102	-0.0576	0.1222	0.1230	0.0173	0.0061	-0.0063
18	-0.1214	0.0876	0.0557	0.3712	0.1805	0.3010	-0.1228	0.0345	0.4327	0.0650	-0.0128	0.0002	0.0000	0.0000
19	-0.1149	-0.2474	-0.1257	0.0622	0.1647	-0.0396	-0.1398	0.0712	-0.0989	0.3089	-0.2251	-0.1249	-0.0235	0.0048
20	-0.1061	0.1557	-0.0321	-0.3734	0.3504	-0.0553	-0.3950	0.1534	0.0035	-0.0146	0.0019	-0.0015	-0.0003	0.0000
21	-0.1359	0.0282	0.0238	0.0432	-0.0205	-0.0703	0.0368	0.0089	-0.0538	-0.0358	-0.1793	0.0047	0.0054	-0.0091
22	-0.1351	0.0389	0.0290	0.0380	-0.0224	-0.0714	0.0350	0.0104	-0.0609	0.1581	0.1230	0.0221	0.0063	-0.0050
23	0.2703	0.1115	-0.2770	0.1306	-0.2443	-0.0706	-0.2027	-0.0443	0.0015	0.0009	-0.0002	0.0525	-0.3938	-0.4471
24	0.2709	0.1106	-0.2755	0.1326	-0.2497	-0.0726	-0.2093	-0.0339	0.0012	-0.0008	0.0002	-0.0528	0.3935	0.4393
25	0.3152	0.0273	-0.1115	0.0021	0.0276	0.0892	0.3016	0.5107	-0.0142	-0.0004	0.0000	-0.0008	0.0037	-0.0025
26	0.2077	0.2004	-0.3678	-0.2457	0.3873	0.0419	-0.0460	-0.0907	0.0113	-0.0936	0.0211	0.5312	0.0964	-0.0140
27	0.2969	-0.1070	0.1666	0.0899	-0.2251	-0.0800	-0.2638	0.0894	-0.0009	-0.0047	0.0006	0.0946	-0.4795	-0.0006
28	0.2174	0.1638	-0.3610	0.0074	0.1116	0.1004	0.4105	-0.3310	0.0047	0.1025	-0.0217	-0.5311	-0.0967	0.0244
29	0.2967	-0.1086	0.1701	0.0864	-0.2178	-0.0781	-0.2589	0.0773	-0.0005	-0.0033	0.0005	-0.0926	0.4837	-0.0010
30	0.2487	-0.1635	0.3111	-0.0975	0.1808	0.0769	0.2237	0.1664	-0.0041	-0.0004	0.0001	0.0708	-0.3123	0.5460
31	0.2482	-0.1634	0.3113	-0.0985	0.1832	0.0767	0.2224	0.1502	-0.0036	0.0008	-0.0001	-0.0708	0.3107	-0.5543
32	0.2190	-0.1686	0.3368	-0.1019	0.1674	-0.0174	-0.1239	-0.6919	0.0233	0.0092	-0.0008	-0.0021	-0.0074	0.0110
